# Body Size Predicts Echolocation Call Peak Frequency Better than Gape Height in Vespertilionid Bats

**DOI:** 10.1038/s41598-017-00959-2

**Published:** 2017-04-11

**Authors:** Jeneni Thiagavel, Sharlene E. Santana, John M. Ratcliffe

**Affiliations:** 1 Department of Ecology and Evolutionary Biology, University of Toronto, Toronto, Canada; 2 Department of Biology and Burke Museum of Natural History and Culture, University of Washington, Seattle, USA

## Abstract

In most vocalizing vertebrates, lighter animals tend to produce acoustic signals of higher frequency than heavier animals. Two hypotheses propose to explain this negative relationship in vespertilionid bats: (i) mass-signal frequency allometry and (ii) emitter-limited (maximum gape) signal directionality. The first hypothesis, that lighter bats with smaller larynges are constrained to calls with higher frequencies, is supported at the species level. The second hypothesis proposes that in open space, small bats use higher frequencies to achieve narrow sonar beams, as beam directionality increases with both emitter size (maximum gape) and signal frequency. This hypothesis is supported within a comparative context but remains untested beyond a few species. We analyzed gape, body mass, and echolocation data under a phylogenetic comparative framework to test these hypotheses, and considered forearm length as both a proxy for wing design and an alternative measure of bat size. Controlling for mass, we found no support for the directionality hypothesis. Body mass and relative forearm length were negatively related to open space echolocation call peak frequency, reflecting species-specific size differences, but also the influence of wing design and preferred foraging habitat on size-independent species-specific differences in echolocation call design.

## Introduction

**Figure 1:**
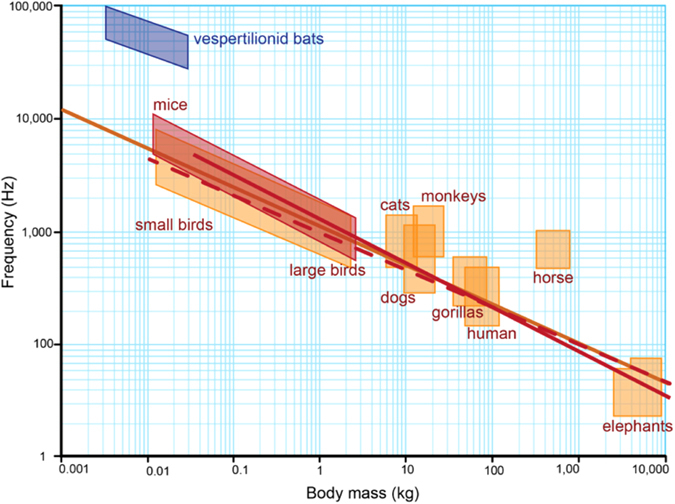
Body mass versus vocalization frequency for a range of vertebrates. Figure made by Sara Vukson using data presented in Jones^2^ and Fletcher^43^.

Many bat species emit echolocation calls of extraordinarily high frequencies. The trident bat, *Cleotis percivalis* (Hipposideridae), emits calls with a carrier frequency of 212 kHz, the highest frequency pure tone documented from the natural world^1^. However, this is the upper limit and most echolocating bats produce frequency modulated calls with peak frequencies (PF, frequency of maximum energy) between 20 and 60 kHz, still well above the upper limit of adult human hearing. Across vespertilionids (the most species rich bat family, ~420 species) echolocation call PFs range from 10 to 150 kHz, and mass from 2 to 60 grams. Size-signal allometry has been hypothesized to explain this intra-familial variation in echolocation call PFs^2^, resembling the negative relationship observed between body size and acoustic signal frequency in birds and anurans^3, 4^ (Fig. 1). Across groups, this trend has been explained as the consequence of a universal scaling law, where frequency is inversely proportional to size^5^. That is, the minimum frequency that can be produced decreases as the size of the sound producing structure increases^5^. Thus, small vespertilionids are expected to be constrained to high frequencies by their relatively smaller bodies and larynges^2, 6^.

**Figure 2:**
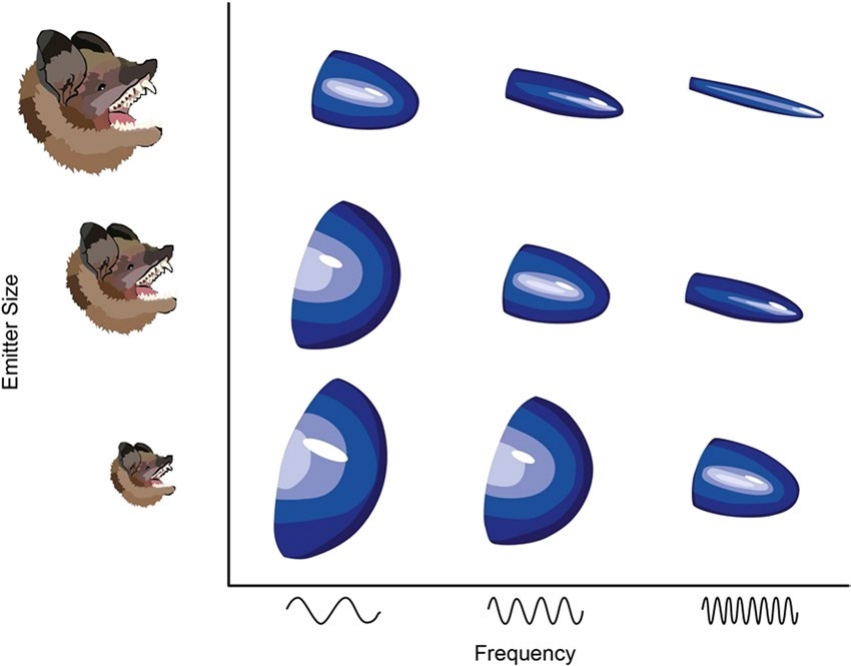
Relationship between directionality, emitter size, and frequency in vespertilionid bats. Directionality of the biosonar beam increases with emitter size and frequency. Figure remade by Sara Vukson from Jakobsen *et al*.^8^.

An alternative hypothesis, sonar beam directionality, has recently gained attention as an overlooked explanation for (i) the production and use of high frequency sounds and (ii) the negative relationship between size and call frequency in vespertilionid bats^7, 8^. Vesper bats emit echolocation calls through their mouths, and emitter size (i.e. gape) and PF are both positively related to call directionality^9^ (Fig. 2). All else being equal, more directional (i.e. long, narrow) beams have more energy focused along the acoustic axis, increasing sonar range while minimizing potentially distracting off-axis echoes^10, 11^. Vespers unable to produce wide gapes (i.e. those with small mouths and/or short snouts), which would otherwise produce low directionality calls, have been argued to use very high frequency calls to achieve highly directional sonar beams while hunting in open space^8^. For vespers, it has been suggested that maximum gape may better predict species-specific PF than body mass^8^.

Neither of these proposed morphological correlates of echolocation call frequency has been assessed independently of the other, nor has either been considered within a phylogenetic comparative framework. We therefore set out to test the explanatory power of mass and gape on echolocation call PF in vesper bats under current phylogenetic hypotheses. We also consider forearm length, a size measure perhaps more precise than mass (which in bats can vary greatly over the course of a day and across the seasons)^12, 13^. Forearm length is also a relative indicator of bat flight style and habitat use^14^, and thus can provide insight into acoustic specialization across preferred habitats. We predicted that the two proxies of body size (i.e. mass and forearm length) would be strong, independent predictors of PF due to size-signal allometry (as observed in non-echolocating vocalizing vertebrates) but that gape height (when corrected for mass) would not influence PF as strongly.

## Results

We found significant phylogenetic signal in all variables (mass: λ = 0.551, *p* *=* < 0.001; forearm length: λ = 0.57, *p* < 0.001; gape height: λ = 0.49, *p* < 0.001; peak frequency: λ = 0.91, *p* < 0.001), and a lambda model provided the best fit for the data (Table 1). Using phylogenetic generalized least squares (PGLS) by restricted maximum likelihood (REML), we found that peak frequency (PF) scaled negatively with mass, forearm length, and gape height (mass: b = −0.21 ± 0.04, t = −5.08, p < 0.001, λ = 0.84, AIC = −9.69, d.f. = 85; FA: b = −0.65 ± 0.13, t = −4.96, p < 0.001, λ = 0.86, AIC = −18.7, df = 68; GH: b = −0.52 ± 0.13, t = −4.1, p < 0.001, λ = 0.92 AIC = −3.56, d.f. = 85). Mass-corrected PF also scaled negatively and significantly with mass-corrected forearm length and negatively with mass-corrected maximum gape height but this relationship was not significant (FA: b = −0.47 ± 0.17, t = −2.9, p = 0.005, λ = 0.85, AIC = −21.39, d.f. = 68; GH: b = −0.14 ± 0.2 t = 0.2, p = 0.46, λ = 0.85, AIC = −10.19, d.f. = 85). Similar to mass-corrected gape, forearm-corrected PF also scaled negatively with forearm-corrected gape height but the relationship was not significant (b = −4.8e-02 ± 2.01e-01, t = −0.2, p = 0.81, λ = 0.86, AIC = −18.71, d.f. = 68, *R* ^2^ = 0.01).

**Table 1:** The fit of different evolutionary models (AICc values are shown).

Trait	Brownian Motion	Lambda	Ornstein-Uhlenbeck	Early Burst
Mass	227.97	145.12	165.56	230.12
Forearm length	51.14	−36.55	−23.83	53.33
Gape height	37.31	−44.49	−34.67	39.46
Peak frequency	1936	10.44	20.54	21.51

## Discussion

Controlling for phylogeny, we found that in open space vespertilionids’ call peak frequencies decrease significantly with body mass, forearm length, and maximum gape height. After correcting for mass, forearm length remains a significant, negative predictor of peak frequency, but maximum gape does not. These findings substantiate earlier results^2^ and suggest that small vesper species are constrained to high frequencies by their smaller bodies and larynges^2, 6^. In other words, when accounting for the effects of size and shared ancestry, we find support for the hypothesis that peak frequency decreases with body size in vespertilionid bats (i.e. the mass-signal frequency allometry hypothesis) and little to no support for the emitter-limited (maximum gape) signal directionality hypothesis.

However, even though peak frequency decreases significantly with the two proxies of body size, this still does not account for the incongruence between body mass and signal frequency relative to non-echolocating mammals (Fig. 1). Vespertilionids vocalize at much higher frequencies than do similarly sized non-echolocating mammals, despite having much larger larynges^15^ (Fig. 1). Thus, although body size appears to be an important predictor of peak frequency, an allometric relationship alone is insufficient to explain their signal diversity^7, 8^. Why the larynges of echolocating bats are larger than similar sized mammals and why, despite this, they call at much higher frequencies than these animals, is not entirely clear. Bats are louder than most mammals^16, 17^, and larger larynges may be required to produce these loud sounds. Additionally, the unusually high frequencies emitted by laryngeal echolocating bats have been attributed to specialized vocal membranes found atop the vocal folds, only the latter are typical of other non-echolocating mammals (reviewed in Neuweiler^18^; Ratcliffe *et al*.^19^). More comparative research into echolocation call production mechanisms in vespertilionids should provide better insight into species-specific echolocation call frequency composition.

After controlling for species’ size, we found no support for the emitter-limited directionality hypothesis. Mass and absolute gape height had both appeared to be good predictors of peak frequency^2, 8^, but in our study gape did not remain so after size-correction within a phylogenetic context. Thus, while directionality had been put forth as a potentially better predictor of open space PF than body size^8^, we do not find support for the directionality hypothesis. That is, when using 86 vespertilionid species and phylogenetically informed mass residuals (to account for common ancestry and size), the once apparent relationship between PF and gape essentially disappeared. Therefore, a single convergent open space field of view may not apply to all vespertilionids. Bats which preferentially hunt in cluttered habitat, for instance, may be constrained to relatively broad beams even when flying in open space as a result of other, perhaps competing, demands on overall call design. Other factors may also contribute to shape situation-specific optimal call design for bats. For instance, facial features such as nose leaves and exaggerated lip and tongue morphologies can affect sound emission patterns^20^. Indeed, facial musculature has recently been shown to alter beam formation in free-tailed bats^21^. Taking these various features into account may provide further insight into beam shape and size by echolocating vespertilionid bats.

Because small bats tend to call at higher frequencies than larger bats, it had once been thought that such high frequencies were specialized to detect prey of a preferred size class. This idea has since been largely rejected. First, the majority of echolocating bats call at frequencies 3 or more times higher than should be necessary to detect the smallest prey found in their diet^7, 22^. Second, while larger bats do take larger prey than smaller bats, they can also detect and intercept small prey^23^. Instead, it is small bats that appear to be limited in the prey sizes they can take, as a result of handling effort and perhaps interspecific competition, not sensory system constraints^23^. Still, consideration of diet is important. For instance, some bats like the ~16 g spotted bat, *Euderma maculatum*, use call designs that circumvent insect defenses. *E. maculatum* calls at ~10.5 kHz, a PF (much lower than predicted by body size) and eats mostly eared moths that are mostly deaf to frequencies below 15 kHz^24^. Further, diet relates to jaw morphology: bats with long, gracile jaws are limited to soft bodied insects while the jaws of those species that can consume harder shelled prey (e.g., beetles) are relatively shorter and stronger^25^. More rigorous accounting of the relationships between diet, gape, size, call parameters, and beam directionality across laryngeal echolocating bats may provide further insight into active sensing and prey selection.

In keeping with the theme of ecological impacts, forearm length is a significant predictor of peak frequency, both before and after size correction. Interestingly, PF decreased more with absolute and mass-corrected forearm than it did with mass (i.e. had a steeper negative slope), suggesting that, for a given mass, bats with longer forearms use lower peak frequency echolocation calls in open space. Relative forearm length is also a proxy for different wing morphologies, which relate to different foraging ecologies. Insectivorous bats with relatively short forearms tend to have short, broad wings with low wing loadings^14, 26, 27^ and aspect ratios^14, 28–30^. It has been suggested that this wing design is well suited for slow, maneuverable flight in cluttered habitat, but may be disadvantageous for successful prey capture in open space^14, 29^. Bats with relatively long forearms, conversely, tend to have long, narrow wings of high wing loading and aspect ratio. This wing design demands fast, agile flight and may be best suited for aerial capture in open spaces^14^. All else being equal, in open space, bats that have long narrow wings are expected to use echolocation calls with lower PFs than those species with short, broad wings^29, 30^. Our results support this prediction. Such calls maximize an echolocating bat’s detection range^31–33^. The higher peak frequency calls of slower, more maneuverable fliers should translate into more precise information for object ranging and resolution^8, 32^.

## Materials and Methods

### Data assembly

We photographed, in lateral view, the cranium and the mandible of vespertilionid bat species at the Royal Ontario Museum (ROM) in Toronto, Canada using a Nikon D40x digital SLR camera. We also photographed skins (≤4 individuals/species) to obtain forearm measurements. Whenever possible, we selected skins and skulls from the same specimen, and included the same number of males and females per species. We imported all photos into Image J v. 1.49^34^ to measure skull characteristics and forearm length 9 times (3 hypothesis-blind assistants, 3 times each) to obtain a single mean value for each measure for each species.

**Figure 3:**
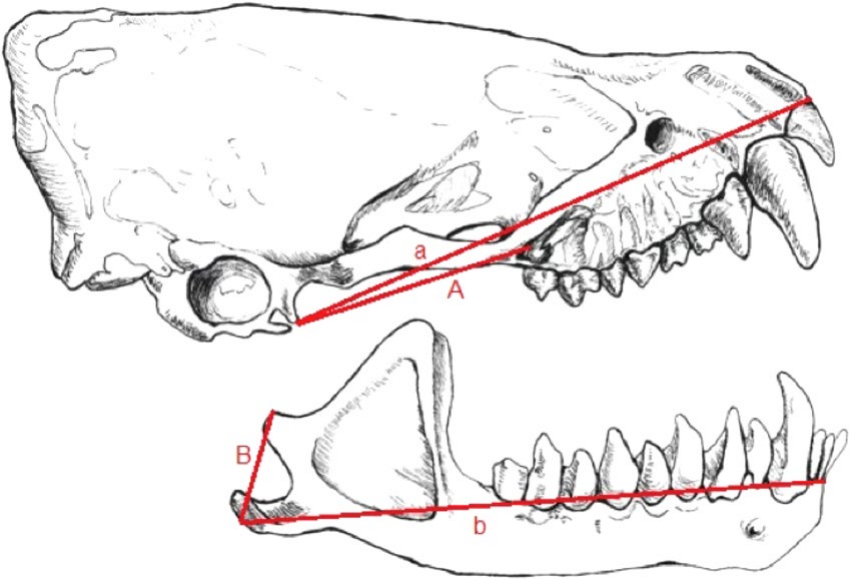
Distances used to measure maximum gape height. Maximum gape height was estimated using the distances between the posterior-most point of the temporomandibular joint and the anterior-most point at the upper incisors (a), lower incisors (b), and origin (A) and insertion (B) of the superficial masseter to estimate the maximum gape for each individual. Figure made by Sara Vukson.

We measured the distances between the posterior-most point of the temporomandibular joint and the anterior-most point of the upper incisors (a), lower incisors (b), and origin (A) and insertion (B) of the superficial masseter to estimate the maximum gape for each individual (Fig. 3). We used a reported gape angle of 90° and A:B ratio of 2.1, as reported for *Myotis lucifugus* ^35^, to estimate maximum gape height (GH) using equation (1).

1 $$Gape\,Heigh{t}_{species}=\sqrt{{a}^{2}+{b}^{2}-2ab\ast \,\cos (90^\circ \ast \frac{A/{B}_{species}}{A/{B}_{lucifugus}})}$$

(as described by Herring and Herring^36^, and modified by Jakobsen and colleagues^8^; Fig. 3). This method of estimating gape height is robust for vespertilionids and other primarily insectivorous bat families^37^. For a given frequency, emitter size primarily determines signal directionality in vespertilionid bats^38^. This relationship among emitter size, maximum estimated gape, actual call emission gape, and call PF and directionality have been confirmed in *Myotis daubentonii* ^8, 10^.

We used mass and forearm length as proxies for body size. We measured maximum forearm length as the length between the elbow and wrist^39^. Mass (g), species-specific open-space echolocation call peak frequency (PF; frequency with the maximum energy in first ‘harmonic’ in kHz) were obtained from a single, comprehensive review of the literature^40^. For raw data see Supplementary Figure S1. The data for all continuous variables used in this study were not normally distributed and were thus log-transformed in subsequent analyses.

### Statistical analyses

**Figure 4:**
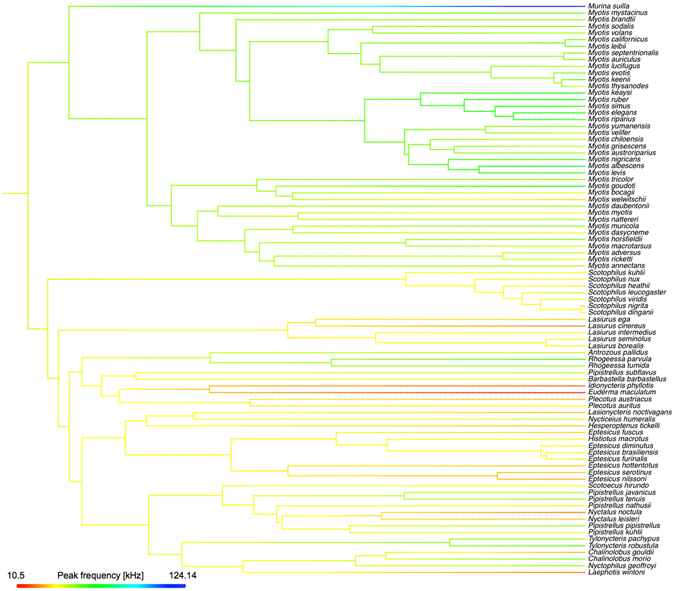
A phylogeny (Shi and Rabosky)^41^ showing peak frequency (kHz) mapped along the branches of the tree using Maximum Likelihood.

We found a significant phylogenetic signal in all variables (Pagel’s λ not significantly different from 1; see Results). Thus, we conducted all subsequent statistical analyses using a lambda evolutionary model and a pruned version of a recent, time-calibrated, molecular phylogeny^41^ (Fig. 4). For these analyses, we used all vespertilionid species (i) with call parameters in Collen^40^, (ii) included in the Shi and Rabosky^41^ phylogeny, and (iii) for which the ROM had at least one intact adult skull or taxidermied specimen. This resulted in 86 species (260 specimens) with mass and gape data, and 69 species (200 specimens) with forearm data.

We used phylogenetic generalized least squares (PGLS) regressions by restricted maximum likelihood (REML) to test the relationship between peak frequency and (i) body mass, (ii) forearm length, and (iii) maximum gape height, respectively. However, since gape height is significantly and positively correlated with body size metrics (b = 0.263 ± 0.02, t = 12.09, p < 0.001, d.f. = 85, *R* ^2^ (Phylogenetic Independent Contrasts regression) = 0.63, we ran re-ran the analyses using size-corrected gape (residuals on mass or forearm length via PGLS regressions; Revell^42^)). Similarly, since PF was significantly negatively correlated with body size, we used size-corrected PF as well.

## Electronic supplementary material

Supplementary Table
