# Supplementary material for: Body Size Predicts Echolocation Call Peak Frequency Better than Gape Height in Vespertilionid Bats
Source: Sci Rep. 2017 Apr 11;7:828. doi: 10.1038/s41598-017-00959-2 (PMC5429766; doi:10.1038/s41598-017-00959-2)
Supplement: Supplementary file 1 — Supplementary Table [file 41598_2017_959_MOESM1_ESM.pdf]

Supplementary Table S1 for *NPG Scientific Reports* research article:

Body Size Predicts Echolocation Call Peak Frequency Better than Gape Height in Vespertilionid Bats

Authors:

Jeneni Thiagavel, Sharlene E. Santana, and John M. Ratcliffe

SupplementaryTable S1. Size and call parameters for the vespertilionid species used in the study (*N*=86)

Species	Peak Frequency (kHz)	Mass (g)	Gape height (mm)	Forearm Length (mm)
Antrozous pallidus	44.76	22.24	20.9	53.93
Barbastella barbastellus	35.33	8.31	14.02	37.37
Chalinolobus gouldii	29.39	14.24	16.44	
Chalinolobus morio	52.92	8.91	11.5	40.75
Eptesicus brasiliensis	34.86	9.2	16.92	39.1
Eptesicus diminutus	40.85	5.99	14.59	
Eptesicus furinalis	42.84	7.7	16.92	37.88
Eptesicus fuscus	32.55	17.49	21.97	45.86
Eptesicus hottentotus	30.88	30.33	21.72	47.57
Eptesicus nilssoni	28.55	10.72	16.61	39.07
Eptesicus serotinus	31.17	23.09	25.33	52.8
Euderma maculatum	10.5	16.17	14.08	50.29
Hesperoptenus tickelli	30.69	16.3	21.68	56.43
Histiotus macrotus	35.84	11	17.94	48.1
Idionycteris phyllotis	20.37	12.13	13.88	47.02
Laephotis wintoni	23.08	6.1	17.93	
Lasionycteris noctivagans	**30.19**	11.02	17.93	40.79
Lasiurus borealis	40.95	12.33	13.63	37.02
Lasiurus cinereus	23.9	27.06	17.26	47.22
Lasiurus ega	32.2	12.2	15.45	
Lasiurus intermedius	36.18	22.96	17.37	49.82
Lasiurus seminolus	40.13	9.88	12.18	43.15
Murina suilla	124.14	4	15.18	
Myotis adversus	46	10.41	13.6	
Myotis albescens	73.02	5.69	13.45	34.68
Myotis annectans	47.51	9.75	17.27	35.52
Myotis auriculus	49.6	38.04	17.93	34.54
Myotis austroriparius	48.23	7.35	15.82	39.36
Myotis bocagii	41.26	7.93	17	34.59
Myotis brandtii	49.27	5.3	16.56	34.3
Myotis californicus	51.44	4.39	13.47	
Myotis chiloensis	43.4	8.41	14.12	
Myotis dasycneme	39.43	15.16	18.31	
Myotis daubentonii	46.98	7.63	14.64	37.69
Myotis elegans	66.28	4.21	14.06	33.5
Myotis evotis	52	6.91	16.07	36.77
Myotis goudoti	64.37	5.56	16.14	38.26
Myotis grisescens	44.52	10.84	17.98	43.68
Myotis horsfieldii	51.21	6.05	15.89	29.43
Myotis keaysi	62.93	5.45	14.79	36.81
Myotis keenii	59.67	6.51	14.74	35.47
Myotis leibii	55.78	5.22	14.31	31.08
Myotis levis	57.74	5.49	15.7	36.12
Myotis lucifugus	47.39	7.8	14.99	38.38
Myotis macrotarsus	42.82	12.64	21.75	43.08
Myotis muricola	48.69	4.8	13.52	
Myotis myotis	35.45	25.59	27.35	
Myotis mystacinus	52.66	7.61	15.39	38.86
Myotis nattereri	46.95	7.25	15.87	40.36
Myotis nigricans	56.2	5.53	13.58	34.44
Myotis ricketti	40.52	26.19	24.35	37.32
Myotis riparius	57.68	4.57	14.02	35.62
Myotis ruber	62.49	4.99	15.1	37.85
Myotis septentrionalis	49.93	5.4	16	35.91
Myotis simus	57.74	8.1	13.84	36.43
Myotis sodalis	51.42	7.15	14.8	38.13
Myotis thysanodes	36.52	8.49	16.53	42.29
Myotis tricolor	49.12	13.67	20.55	50.45
Myotis velifer	43.44	9.82	17.75	43.33
Myotis volans	46.39	8.71	14.77	40.25

Myotis welwitschii	32.66	15.88	22.6	
Myotis yumanensis	51.47	5.15	14.11	31.91
Nyctalus leisleri	30.67	12.47	16.23	33.96
Nyctalus noctula	22.86	28.48	22.26	49.58
Nycticeius humeralis	42.82	9.12	14.07	33.57
Nyctophilus geoffroyi	49.77	8.2	17.5	
Pipistrellus javanicus	47.51	4.92	12.97	
Pipistrellus kuhlii	41.16	6.07	14.53	30.84
Pipistrellus nathusii	41.4	7.44	13.31	33.93
Pipistrellus pipistrellus	47.61	5.3	13.79	33.58
Pipistrellus subflavus	45.95	5.74	12.47	32.62
Pipistrellus tenuis	48	3.48	13.29	
Plecotus auritus	36.39	8.19	13.5	39.99
Plecotus austriacus	29.66	6.75	13.23	40.83
Rhogeessa parvula	57.57	4.37	11.11	27.23
Rhogeessa tumida	48.65	4.58	12.62	28.83
Scotoecus hirundo	36.86	8.45	16.95	34.9
Scotophilus dinganii	36.38	25.12	19.44	53.04
Scotophilus heathii	34.81	36.13	21.99	
Scotophilus kuhlii	38.94	20.31	17.53	51.32
Scotophilus leucogaster	46.27	20.24	18.01	49.46
Scotophilus nigrita	32.85	60	28.07	80.78
Scotophilus nux	36.09	30	21.55	53.99
Scotophilus viridis	40.1	19.81	17.01	46.17
Tylonycteris pachypus	48.95	4.1	14.2	
Tylonycteris robustula	55	7.98	15.07	23.14
